# The Oncopig Cancer Model as a Complementary Tool for Phenotypic Drug Discovery

**DOI:** 10.3389/fphar.2017.00894

**Published:** 2017-12-05

**Authors:** Natalia V. Segatto, Mariana H. Remião, Kyle M. Schachtschneider, Fabiana K. Seixas, Lawrence B. Schook, Tiago Collares

**Affiliations:** ^1^Biotechnology Graduate Program, Molecular and Cellular Oncology Research Group, Laboratory of Cancer Biotechnology, Technology Development Center, Federal University of Pelotas, Pelotas, Brazil; ^2^Department of Radiology, University of Illinois at Chicago, Chicago, IL, United States; ^3^Department of Animal Sciences, University of Illinois at Urbana–Champaign, Champaign, IL, United States

**Keywords:** PDD, animal model, swine, Oncopig cancer model, cancer

## Abstract

The screening of potential therapeutic compounds using phenotypic drug discovery (PDD) is being embraced once again by researchers and pharmaceutical companies as an approach to enhance the development of new effective therapeutics. Before the genomics and molecular biology era and the consecutive emergence of targeted-drug discovery approaches, PDD was the most common platform used for drug discovery. PDD, also known as phenotypic screening, consists of screening potential compounds in either *in vitro* cellular or *in vivo* animal models to identify compounds resulting in a desirable phenotypic change. Using this approach, the biological targets of the compounds are not taken into consideration. Suitable animal models are crucial for the continued validation and discovery of new drugs, as compounds displaying promising results in phenotypic *in vitro* cell-based and *in vivo* small animal model screenings often fail in clinical trials. Indeed, this is mainly a result of differential anatomy, physiology, metabolism, immunology, and genetics between humans and currently used pre-clinical small animal models. In contrast, pigs are more predictive of therapeutic treatment outcomes in humans than rodents. In addition, pigs provide an ideal platform to study cancer due to their similarities with humans at the anatomical, physiological, metabolic, and genetic levels. Here we provide a mini-review on the reemergence of PDD in drug development, highlighting the potential of porcine cancer models for improving pre-clinical drug discovery and testing. We also present precision medicine based genetically defined swine cancer models developed to date and their potential as biomedical models.

## The Return of Phenotypic Drug Discovery

The pharmaceutical industry has increasingly invested in the research and development (R&D) of new potential drugs and has doubled these investments over the past 15 years, from $26.0 billion in 2000 to an estimated $58.8 billion in 2015 (PhRMA, 2016). Surprisingly, companies have produced on average less than one new drug per year since 1950, indicating that no strategy employed by pharmaceutical companies over this time period has increased their ability to discover and bring new drugs to market (Munos, 2009). Analysis of the drugs approved by the FDA between 1999 and 2008 indicates more first-in-class drugs have been approved using phenotypic screening assays than target-based approaches (Swinney and Anthony, 2011).

Phenotypic drug discovery (PDD) consists of screening potential compounds in either *in vitro* cellular (Mosmann, 1983) or *in vivo* animal models (Giacomotto and Segalat, 2010) to identify compounds resulting in a desirable phenotypic change. On the other hand, target-based drug discovery (TDD) relies on the identification of a target of interest believed to be “disease-modifying” and therefore related to a particular disease. The genomic era revolutionized the screening of new compounds *in vitro* and *in vivo* through the rise of comparative genomics, genome editing, and the consecutive emergence of TDD approaches. Even though TDD has several advantages, such as confirmation of the relevance of a target for a given disease, this approach has not effectively translated into the approval of new drugs as expected. Some researchers believe that one of the reasons for the decrease in drug discovery and innumerous failed clinical trials in the last 25 years may be due to the extensive use of TDD in the past decades (Sams-Dodd, 2005, 2013; Hellerstein, 2008). Therefore, researchers and pharmaceutical companies are once again embracing PDD as a way to improve development and screening of new effective therapeutics (Lee and Berg, 2013; Zheng et al., 2013; Warchal et al., 2015).

## Pdd In Cancer Drug Discovery

Oncology is a huge market for pharmaceutical industries, and has grown to be the largest therapeutic area in terms of number of projects, investments in research and development (R&D), and number of clinical trials (Arrowsmith, 2012). However, discovering and approving new efficient cancer therapies is a challenge (Hoelder et al., 2012) mainly because cancer is a highly heterogeneous disease with multiple mechanisms of action. The hallmarks of cancer indicate that the transformation of normal cells into malignant cancer cells is a multistep process reflecting genetic alterations affecting six physiological processes. These processes include self-sufficient growth signaling, insensitivity to growth-inhibitory (antigrowth) signals, evasion of programmed cell death (apoptosis), limitless replicative potential, sustained angiogenesis, and tissue invasion and metastasis (Hanahan and Weinberg, 2000).

The majority of the cancer drugs developed in the past decades have been discovered using target-based approaches (Swinney and Anthony, 2011). This is expected, as our knowledge of the molecular basis of cancer is growing (Garraway and Lander, 2013) and as a consequence new cancer therapeutics are focused on targets known to be related to the above-mentioned multistep processes. For example, as kinases play key roles in signal transduction and regulation of a range of cellular activities, kinase inhibitors represent 21 out of the 29 approved drugs developed using TDD between 1999 and 2008 (Zhang et al., 2009; Swinney and Anthony, 2011).

It is important to highlight that when researchers focus only on known targets for drug discovery, they lose the opportunity to identify potential drugs that may have different—and new—targeted mechanisms of action. Between 1999 and 2008, 19 of the approved cancer therapeutics were discovered using some level of phenotypic screening (Swinney and Anthony, 2011). Among them are the thalidomide analog Lenalidomide, for which the selection of second-generation analogs with anticancer activity was conducted using only phenotypic assays (Shortt et al., 2013). In addition, the observation that dimethyl sulfoxide (DMSO) caused growth arrest (Friend et al., 1971; Takase et al., 1992; Teraoka et al., 1996) and terminal differentiation of transformed cells (Friend et al., 1971) led researchers to test other polar, small-molecule solvent species for antitumor activity, resulting in the discovery of the histone deacetylase inhibitor Vorinostat (Marks and Breslow, 2007). Furthermore, Romidepsin, an anticancer agent approved for the treatment of cutaneous T-cell lymphoma was identified through phenotypic screening of microbial metabolites in tumor cell lines (Ueda et al., 1994).

In general, PDD remains a crucial approach in selecting, validating, and developing potential cancer drugs, even though it represents a minority of the investigational and recently approved oncology treatments (Moffat et al., 2014). PDD screening is only the first step in the long and expensive journey of drug discovery. The next stage, represented by animal experimentation in pre-clinical trials, must validate the promising results obtained in the initial screening. Therefore, both steps are complementary and equally important.

## Importance of Animal Models In Drug Discovery

Animal models are essential tools in the drug development process. Due to ethical and regulatory issues, it is necessary to test new biomedical products in animal models during the pre-clinical phase before initiating human experimentation to evaluate efficacy, toxicity, and safety. However, many drugs that display promising results in pre-clinical animal studies do not produce the same response in humans (Mak et al., 2014). This is especially true for oncology due to difficulties in mirroring the heterogeneity and complex tumor characteristics in animal models (Huszthy et al., 2012; van Marion et al., 2016). Disregarding the approach used for drug discovery (TDD or PDD), there are several examples of candidate therapeutics failing in clinical trials even though they showed promising results in previous phases of drug development (Williams, 2013, 2015). In fact, about 85% of therapies tested in clinical trials fail (Ledford, 2011), with cancer therapeutics representing the largest proportion of these failures (Arrowsmith, 2011). Only 5% of agents that demonstrate anticancer activity in preclinical phases are approved after demonstrating sufficient efficacy in phase III testing (Hutchinson and Kirk, 2011).

One of the key takeaways from these failures is the need for improvements in the early steps of drug discovery, specifically in the use of adequate animal models in pre-clinical trials. Suitable animal models are crucial for the continued validation and discovery of new drugs, as compounds displaying promising results in phenotypic *in vitro* cell-based and *in vivo* small animal model screenings often fail in clinical trials.

Rodents are the most widely used platform for tumor pre-clinical screening and biological models in general. Some of their characteristics, such as small size, inexpensiveness, well-known genetics, and their ability to be easily genetically modified make them a standard tool for evaluating novel therapeutics. However, they are actually poor models for many human diseases (Seok et al., 2013; Burns et al., 2015), including cancer (de Jong and Maina, 2010). Consequently, difficulties translating the results obtained in pre-clinical studies to the clinical realm are quite common when rodents are chosen as the biological model. One example is the phase II clinical trial of IPI-926 (Saridegib) in patients with advanced chondrosarcoma. The trial was stopped early because no relevant effects were observed in humans treated with Saridegib compared to the placebo group (Wagner et al., 2013), even though medulloblastoma mice models treated with IPI-926 demonstrated a fivefold increase in survival (Lee et al., 2012). This work demonstrates the need for more relevant animal models as complementary tools for cancer drug discovery.

Even though genetically engineered mouse models are important tools for studying mechanisms associated with cancer biology (Jeong, 2016; Jiang and Yu, 2017; Perez-Guijarro et al., 2017) and represent improved models compared to wild mice models, they still have several limitations. We believe that the use of a suitable large animal model of cancer would improve the results of PDD for cancer therapeutics.

## Pigs As Biological Models

Pigs have proven to be more predictive of therapeutic treatments in humans than rodents (Meurens et al., 2012; Schook et al., 2015a). Pigs provide an ideal platform to study cancer due to their similarities with humans at the anatomical, physiological, metabolic, and genetic levels (**Table 1**). The pig genome sequence was published in 2012, providing insights into their genetic similarities to humans (Groenen et al., 2012) and furthering their acceptance as a large animal biomedical model for human diseases (Prather, 2013; Schook et al., 2015a). In addition to the high homology between the pig and human genome, the swine genome also exhibits highly conserved epigenetic regulation demonstrated by the similar genome wide DNA methylation patterns observed between pigs and humans (Schachtschneider et al., 2015). Regarding their cancer genetics, a previous study using genetically engineered porcine cells showed that swine cells could be transformed by mutated oncogenes and tumor suppressor genes commonly found in human cancers (Adam et al., 2007). Furthermore, these transformed cells were able to form tumors following autologous injection (Adam et al., 2007). Also, normal murine cells can be transformed with fewer mutations (Rangarajan et al., 2004) compared to swine and human cells (Rangarajan et al., 2004; Adam et al., 2007). Together, this work demonstrates that porcine tumorigenic pathways are more similar to human pathways than rodents (**Table 1**).

**Table 1:** Characteristics of murine and swine pre-clinical trial cancer models compared to humans.

	Murine	Humans	Swine
*Size*	Small (around 3000 times smaller than humans)	Large	Large
	Mouse average weight: 20 g	Human average weight: 62 kg	Minipig average weight: 40 kg
*Metabolism (total Cyp450 content)*	Twofold higher compared to humans (Donato et al., 1999)	Approximately 300–450 pmol/mg protein (Snawder and Lipscomb, 2000)	Comparable to humans (approximately 300–450 pmol/mg protein) (Snawder and Lipscomb, 2000; Burkina et al., 2017)
*Metabolism (Cyp3A4)*	Mouse Cyp3a11, Cyp3a16, Cyp3a41a, Cyp3a41b, and Cyp3a44 are less than 80% homologous to the human CYP3A4 nucleotide sequence (Renaud et al., 2011)	N/A	Pig CYP3A22, CYP3A29, CYP3A39, and CYP3A46 are more than 80% homologous to the human CYP3A4 nucleotide sequence (Puccinelli et al., 2011)
*Cancer genetics*	Telomerase activity is found in several mouse tissues (Chadeneau et al., 1995)	Telomerase expression is suppressed in most tissues (Kim et al., 1994)	Telomerase expression is suppressed in most tissues (Pathak et al., 2000)
*Drug administration*	Routes include oral, intravenous (i.v.), intraperitorial (i.p.), intramuscular, intradermal, and intranasal, all of which require smaller volumes compared to humans (Morton et al., 2001)	Routes include oral, i.v., i.p., by inhalation, subcutaneous, intramuscular, epidural, dermal absorption, and transmucosal	Routes include oral, i.v., i.p., by inhalation, subcutaneous, intramuscular, epidural, dermal absorption, and transmucosal – same routes and volumes as humans
*Costs ($ per animal)*	Mutant/genetically modified mice: ±$50-$200;	N/A	Genetically modified swine: ±$1,725;
	Wild-type mice: ±$75		Wild-type swine: ±$575
*Model development time*	Months	N/A	Months

Pigs provide an ideal large animal model for preclinical drug screening due to their metabolic similarities with humans. For instance, pigs have proven to be a suitable model for CYP3A-related drug metabolism. Cytochromes P450 enzymes (CYP) are known for their role in compound metabolism, and the CYP3A subfamily is responsible for metabolizing more than half of all drugs available on the market (Zuber et al., 2002). The swine xenosensor pregnane C receptors also displays high homology to the ones found in humans (Pollock et al., 2007; Gray et al., 2010). In addition, the CYP family receptors and enzymes display similar expression levels in pigs and humans (Nielsen et al., 2017). On the other hand, some rodents (e.g., rats) are not considered good models for CYP3A4 related metabolism due to the dissimilarities with humans in metabolism related to this enzyme, resulting in differential therapeutic metabolization (Martignoni et al., 2006). In contrast, swine models can and have been very useful for pharmacology and toxicology studies (Thorn et al., 2009, 2011).

Another beneficial feature of pigs that makes them a relevant biological model is the fact that they can live up to 10 years. For purposes of cancer drug development, this is especially relevant because it allows for therapeutic testing and posterior monitoring in a pre-clinical platform able to mimic multiple stages of tumor development, progression, invasion, and metastasis, thereby enabling the evaluation of the long-term effects of a variety of compounds. In addition, the large size of the pig is ideal not only for administration of therapeutics in the same manner as in human patients (**Table 1**), but also for the collection of larger volumes of bodily fluids (Helke and Swindle, 2013), allowing blood collection procedures to mirror those performed in humans. We thus hypothesize that the pig’s implementation as an ideal biomedical model for drug discovery could help fill the existing gap between early phenotypic screening of potential new therapeutics, identification of prognostic biomarkers, and testing of beneficial products and devices in human clinical patients.

Genetic engineering allows for the development of transgenic porcine models that recapitulate particular genetic alterations found in human diseases for translational biomedical research purposes. These models can be obtained using several techniques such as microinjection of DNA into the pronuclei of fertilized oocytes, lentiviral transgenesis (LVGT), sperm mediated gene transfer (SMGT), and somatic cell nuclear transfer (SCNT) using genetically modified nuclear donor cells (Aigner et al., 2010). Currently, transgenic pigs are increasingly being accepted as large animal models for several human diseases (Aigner et al., 2010), including neurodegenerative diseases (Kragh et al., 2009), cystic fibrosis (Rogers et al., 2008), cardiovascular diseases (Hao et al., 2006), diabetes mellitus (Renner et al., 2010), and cancer (Flisikowska et al., 2012; Schook et al., 2015b).

## Genetically Defined Swine Models of Cancer

The advancement of genetic engineering techniques combined with knowledge of the pig genome sequence and its similarity to humans (Groenen et al., 2012) make genetic engineering a powerful tool for developing suitable transgenic porcine biological models for cancer drug discovery (Schook et al., 2016).

With the aim of producing a genetically defined porcine cancer model, Schook and collaborators developed the Oncopig Cancer Model (OCM), which contains Cre recombinase inducible mutated tumor suppressor and oncogene transgenes (*TP53^R167H^* and *KRAS^G12D^*, respectively). Exposure to adenoviral vectors encoding Cre recombinase (AdCre) results in cellular transformation in a temporal and spatial manner that closely mimics the spontaneous tumor formation that occurs in humans (Schook et al., 2015b). The intramuscular injection of AdCre into the OCM results in the development of soft-tissue sarcomas (STSs) that display pathological characteristics of human leiomyosarcomas (Schook et al., 2015b). Furthermore, transcriptional profiling of Oncopig STS cell lines and tumors indicates Oncopig STS exhibits altered TP53 signaling, Wnt signaling activation, and signs of epigenetic reprogramming, all of which represent transcriptional hallmarks of human STS (Schachtschneider et al., 2017a). In addition, the transcriptional regulator FOSL1, which was previously identified as a potential human STS therapeutic target, was identified as a master regulator of Oncopig STS (Schachtschneider et al., 2017a).

Two other cancer types have been developed to date in the OCM: hepatocellular carcinoma (HCC) and pancreatic cancer. By isolating and transforming Oncopig hepatocytes via exposure to AdCre *in vitro*, researchers were able to develop Oncopig HCC cell lines expressing *TP53^R167H^* and *KRAS^G12D^* (Schachtschneider et al., 2017b). These cells display histopathological characteristics similar to human HCC and form tumors upon autologous injection into Oncopigs. Furthermore, human HCC transcriptional hallmarks were also observed in Oncopig HCC cells along with conserved gene expression profiles compared to human HCC cell lines (Schachtschneider et al., 2017b). The Oncopig pancreatic ductal adenocarcinoma (PDAC) model is still in development. However, the two most predominant pancreatic cancer histotypes (exocrine and neuroendocrine) have already been developed in this PDAC model via delivery of AdCre into the main pancreatic duct (Diaz et al., 2016).

**FIGURE 1:**
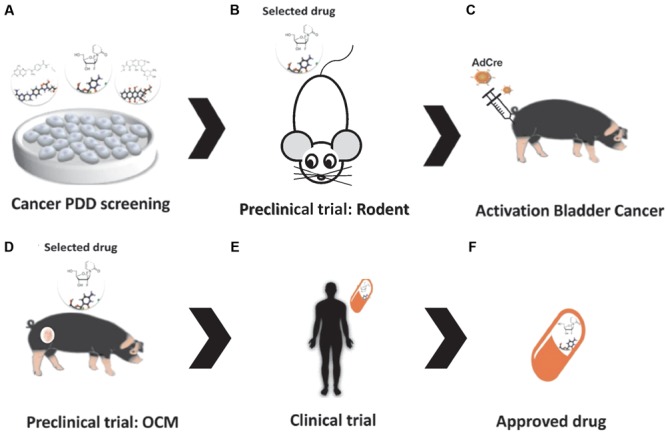
Drug discovery in the Oncopig Bladder Cancer Model. **(A)** Bladder Cancer PDD. **(B)** Pre-clinical trials: *in vivo* drug testing in rodent model. **(C)** Oncopig Bladder Cancer Model: intravesical AdCre injection activates expression of the mutated oncogenes (*TP53^R167H^* and *KRAS^G12D^*), resulting in tumor formation. **(D)** Pre-clinical trial II: *in vivo* drug testing in the Oncopig Bladder Cancer Model. **(E)** Clinical trials. **(F)** Drug selection.

In a recently published article, Schachtschneider et al. (2017c) presented multiple applications of the OCM as an innovative large animal translational oncology platform, ranging from the above-mentioned potential for therapeutic screening and development to its use for developing diagnostic imaging modalities. It also highlights the numerous advantages that swine models have over other commonly used biological models (Schachtschneider et al., 2017c), indicating large animal platforms can serve as predictable models of human therapeutic responses using PDD approaches (**Figure 1**).

Regarding the ethical responsibilities of conducting animal experiments (Perry, 2007) and the three R’s paradigm (Reduce, Replace, and Refine) (Russell and Burch, 1959), the OCM provides an ideal platform capable of discretely inducting localized tumors that can be closely monitored to meet scientific goals while still minimizing the animal’s comorbidities and mortality.

In addition to the OCM, other transgenic swine cancer models have been developed. They include the human familial adenomatous polyposis model in which the *APC* gene was inactivated by introduction of a premature termination codon through electroporation of linearized vector DNA into mesenchymal stem cells followed by SCNT. The animals carrying the *APC^1311^* mutation develop polyps in the colon and rectum after 1 year (Flisikowska et al., 2012). In addition, two models utilizing *TP53* mutations have been developed: a genetically modified pig expressing mutant *TP53^R167H^*, which develops lymphomas and osteogenic tumors in homozygous individuals (Sieren et al., 2014), and *TP53* knockout pigs that develop spontaneous osteosarcomas in older heterozygous animals and multiple large osteosarcomas in 7 to 8-month-old homozygous pigs (Saalfrank et al., 2016). More recently, a genetic model of intestinal cancer was developed using a Flp-recombinase inducible oncogene cassette containing *KRAS^G12D^, cMYC*, and *SV40LT* in addition to a 4-hydroxytamoxifen (4-OHT) activator cassette controlled by an intestinal epithelium tissue-specific promoter. Activation of the oncogene cassette *in vivo* resulted in a duodenal neuroendocrine carcinoma with a lymph node metastasis in the minipig (Callesen et al., 2017). Finally, an attempt to develop a breast cancer model was performed using a recombinant adeno-associated virus (rAAV) mediated *BRCA1* knockout. Unfortunately, these animals died before they were able to demonstrate any relevant phenotypic changes (Luo et al., 2011).

## Future Approaches

We believe the use of porcine models in pre-clinical trials will increase the benefits of PDD. More punctually, the OCM could fulfill the needs of the pharmaceutical industry and academic researchers by providing a model more predictable of therapeutic responses in humans. These higher genetically defined swine cancer models have huge potential to serve as biomedical models in preclinical trials. Since the International Committee on Harmonization requires toxicity testing in two relevant animal species (Food and Drug Administration [FDA], 2010), these models can serve as translational models by testing the efficacy of new therapies that show promising results in small animal PDD screenings before moving to human clinical trials, ultimately decreasing the failure rates of clinical trials (**Figure 1**). In fact, although swine models are more expensive than rodents (**Table 1**), the use of pigs as a second animal model in pre-clinical trials is cheaper than using non-human primates. It can also provide a cost reduction in drug discovery by confirming the trial results before initiating a highly costly human clinical trial.

Furthermore, we hypothesize that intravesical injection of AdCre in the OCM platform could result in tumor formation in the bladder (**Figure 1**); resulting in a highly valuable bladder cancer model in which new compounds and immunotherapies such as recombinant BCG (a promising immunotherapeutic approach for bladder cancer (Begnini et al., 2015) could be tested. Our research group has tested the antitumoral activity of several compounds in bladder cancer cells lines using PDD approaches, including Brazilian red propolis (Begnini et al., 2014) and pyrazoline derivatives (Tessmann et al., 2017). A suitable animal model platform such as the OCM would further advance *in vivo* testing of the most promising compounds selected in these previous studies. To this end, we are currently evaluating the ability of OCM cells to mimic human bladder cancer cell line responses to commercially available therapeutics *in vitro*. We believe that this work will confirm the important role that the OCM can play in PDD of cancer drugs.

## Author Contributions

NS, MR, KS, LS, FS, and TC had an equal participation in writing and approving the present manuscript.

## Conflict of Interest Statement

The authors declare that the research was conducted in the absence of any commercial or financial relationships that could be construed as a potential conflict of interest.
